# Editorial: Harnessing visual computing to revolutionize manufacturing efficiency and innovation

**DOI:** 10.3389/frobt.2026.1831318

**Published:** 2026-03-30

**Authors:** Edmanuel Cruz, Francisco Gomez-Donoso, José Carlos Rangel, Cesar Pinzon-Acosta

**Affiliations:** 1 Centro Regional de Veraguas, Universidad Tecnológica de Panamá, Panamá, Veraguas, Panama; 2 Department of Computer Science and Artificial Intelligence, University of Alicante, Alicante, Spain; 3 Facultad de Ingeniería de Sistemas Computacionales, Universidad Tecnológica de Panamá, Panamá, Panama; 4 Facultad de Ingeniería Mecánica, Universidad Tecnológica de Panamá, Panamá, Panama

**Keywords:** computer vision, machine learning, manufacturing, predictive maintenance, virtual reality

## 1 Introduction: the importance of visual computing in manufacturing

Contemporary manufacturing faces sustained pressure to simultaneously increase productivity, quality, operational safety, and process adaptability. In this context, visual computing has emerged as a high-impact technological pillar by integrating computer vision, machine learning, and advanced perception techniques to automate inspection, monitoring, risk detection, and real-time decision support. This Research Topic was conceived precisely to explore how these technologies can bridge persistent gaps in quality control, defect detection, process optimization, and industrial innovation, while also promoting strategies that facilitate their effective integration into existing manufacturing workflows.

The articles gathered in this Research Topic demonstrate that visual computing should no longer be understood merely as an auxiliary monitoring tool, but rather as an intelligent layer of industrial perception. From automated inspection of weld seams to fire detection, monitoring unsafe behaviors in restricted areas, and reflections on next-generation visual sensors, the works included here show that advances in architectures such as YOLO, transfer learning approaches, and models optimized for real-time inference are redefining industrial efficiency.

## 2 The articles in this collection

The work by Mendieta et al. makes a direct contribution to the core of intelligent manufacturing by addressing the automatic detection of surface discontinuities in Shielded Metal Arc Welding (SMAW). Using a domain-specific dataset of 3,000 images, the authors trained a YOLOv7 architecture, achieving 97% precision and a mAP@0.5 of 94%, with additional evidence of robustness under moderate aspect-ratio variations and the ability to generalize to an external dataset. This study clearly illustrates the value of computer vision for transforming visual inspection, traditionally dependent on human judgment, into a more objective, repeatable, and scalable process for quality assurance in manufacturing.

From a broader perspective on advanced visual perception (Smagulova et al.), provides a comprehensive review of event-based object detection, with a focus on end-to-end perception in autonomous driving. Although their immediate domain is not the industrial plant, their relevance to advanced manufacturing is substantial: event sensors offer dynamic ranges above 100 dB, power consumption typically between 10 and 100 mW, and update rates of approximately 1–10 μ s per event, characteristics that are especially valuable in environments with variable illumination, rapid motion, and energy constraints. By reviewing hardware, event encoding, dense, spiking, and graph-based algorithms, as well as benchmarking criteria, this article broadens the horizon of industrial visual computing toward faster, more efficient, and neuromorphic perception paradigms.

In the field of industrial safety (Deshpande et al.), presents a fire and smoke detection system for indoor and outdoor industrial environments, using a transfer-learning approach based on DetectNet_v2 with ResNet-18 as the backbone. Their proposal is built on a curated dataset of 3,000 images, both real and synthetically augmented, and combines fine-tuning, model pruning, and quantization-aware training to obtain a solution suitable for edge deployment. The results are particularly strong: 95.6% accuracy for fire, 92% for smoke, mAP@0.5 of 94.9%, mAP@0.5:0.95 of 87.4%, a false positive rate of 3.5%, and an average inference time of 42 ms on an RTX GPU. In addition, validation on Jetson platforms reinforces its practical applicability for real-time industrial surveillance scenarios. This work confirms that combining transfer learning and inference optimization can turn artificial vision into an operational layer for prevention and early response in manufacturing.

Complementarily (Deshpande et al.), also explores the detection of unauthorized mobile phone use in restricted manufacturing areas, a problem that combines safety, regulatory compliance, and intellectual property protection. Their system integrates YOLOv8 for real-time detection with dense layers of ResNet-50 for classification, and it was trained on a custom dataset of 2,150 images collected under conditions close to real-world conditions. The results show a mAP50 of 49.5% for the cell phone detection task and a prediction accuracy of 96.03%. Incorporating ResNet-50 improved the model’s precision compared with a version without this reinforcement. Beyond the challenge of detecting small objects, this article is notable for situating visual computing within the domain of human behavior in the plant, where automated perception can also help reduce risks and operational disruptions.

## 3 Conclusion: toward a new industrial efficiency

Taken together, the articles in this Research Topic show that the industrial efficiency of the future will increasingly depend on visual systems capable not only of “seeing,” but also of interpreting, anticipating, and acting within complex operational contexts. Automated weld inspection, intelligent fire surveillance, monitoring of unsafe behaviors, and the evolution toward next-generation visual sensors all converge in the same direction: the construction of industrial environments that are safer, more measurable, and more adaptive.

More importantly, this Research Topic highlights that progress does not depend solely on improving isolated metrics, but on articulating precision, speed, robustness, energy efficiency, and real-world deployability. Architectures from the YOLO family, transfer learning strategies, optimization for edge AI, and new sensory paradigms such as event cameras define a research agenda that goes beyond punctual automation and points toward truly intelligent manufacturing ecosystems. In this sense, this Research Topic not only documents recent advances but also provides a conceptual and methodological foundation for future research to integrate visual computing as a structural component of industrial innovation.

